# A BTP1 prophage gene present in invasive non-typhoidal *Salmonella* determines composition and length of the O-antigen of the lipopolysaccharide

**DOI:** 10.1111/mmi.12933

**Published:** 2015-02-11

**Authors:** Erica Kintz, Mark R Davies, Disa L Hammarlöf, Rocío Canals, Jay C D Hinton, Marjan W van der Woude

**Affiliations:** 1 Centre for Immunology and Infection, Hull York Medical School and the Department of Biology, University of YorkYork, UK; 2 Institute of Integrative Biology, University of LiverpoolUK

## Abstract

*Salmonella* Typhimurium isolate D23580 represents a recently identified ST313 lineage of invasive non-typhoidal Salmonellae (iNTS). One of the differences between this lineage and other non-iNTS *S*. Typhimurium isolates is the presence of prophage BTP1. This prophage encodes a *gtrC* gene, implicated in O-antigen modification. GtrC^BTP^^1^ is essential for maintaining O-antigen length in isolate D23580, since a *gtr*^BTP^^1^ mutant yields a short O-antigen. This phenotype can be complemented by *gtrC*^BTP^^1^ or very closely related *gtrC* genes. The short O-antigen of the *gtr*^BTP^^1^ mutant was also compensated by deletion of the BTP1 phage tailspike gene in the D23580 chromosome. This tailspike protein has a putative endorhamnosidase domain and thus may mediate O-antigen cleavage. Expression of the *gtrC*^BTP^^1^ gene is, in contrast to expression of many other *gtr* operons, not subject to phase variation and transcriptional analysis suggests that *gtrC* is produced under a variety of conditions. Additionally, GtrC^BTP^^1^ expression is necessary and sufficient to provide protection against BTP1 phage infection of an otherwise susceptible strain. These data are consistent with a model in which GtrC^BTP^^1^ mediates modification of the BTP1 phage O-antigen receptor in lysogenic D23580, and thereby prevents superinfection by itself and other phage that uses the same O-antigen co-receptor.

## Introduction

*Salmonella enterica* subspecies *enterica* (*S. enterica*) is responsible for 98% of human infections caused by *Salmonella*. Over 1500 serovars of *S. enterica* are recognized in the Kaufman–White scheme (Grimont and Weill, 2007), which uses differences in the O-antigen of the lipopolysaccharide (LPS) and flagellar H antigen to differentiate between isolates. Over 120 serovars are recognized as having the group O4 O-antigen (Grimont and Weill, 2007), which is characterized specifically by an abequose moiety linked to the mannose with a 1–3 linkage (Reeves, 1993). This is a subgroup of the O12 positive isolates that have an O-antigen subunit backbone consisting of mannose, rhamnose and galactose. *Salmonella enterica* subspecies *enterica* serovar Tyhimurium, hereafter referred to as *S. Typhimurium*, is one of these O4 (previously group B) serovars.

*Salmonella Typhimurium* is a non-host-restricted serovar typically associated with localized gastrointestinal infection in humans. However, there is an epidemic of invasive *S. Typhimurium* infections in Sub-Saharan Africa predominantly associated with the novel multilocus sequence type, ST313 (Kingsley *et al*., 2009; Okoro *et al*., 2012). It is hypothesized that the immunocompromised status of the population allowed evolution of these strains since it can be traced to follow the spread of HIV and is currently not commonly found outside of Africa (MacLennan *et al*., 2010; Okoro *et al*., 2012). Sequencing of a representative ST313 isolate, *S. Typhimurium* D23580, identified several pseudogenes compared with other *S. Typhimurium* isolates. Many of these are shared with the host-restricted *S*. Typhi (Holt *et al*., 2008), which could suggest that D23580 is becoming more host restricted. An additional differentiating feature is the distinct repertoire of five prophage-like elements in the D23580 genome (Kingsley *et al*., 2009). One of these is the BTP1 prophage, and it was recently determined that this prophage carries glycosyltransferase (*gtr*) genes that constitute a putative O-antigen modification operon (Davies *et al*., 2013).

The O-antigen-modifying *gtr* operons have been found in phage genomes, on prophages within *Salmonella* genomes and in the context of phage remnant sequences (Vernikos and Parkhill, 2006; Davies *et al*., 2013). The *gtr* operons described to date consist of three genes: *gtrA*, *gtrB* and *gtrC*. Based on biochemical analysis of P22 mediated O-antigen modification (Makela, 1973) and related *gtr* operons in *Shigella flexneri*, it is hypothesized that GtrA and GtrB add the glucose to a carrier lipid and flip it to the periplasm, where the GtrC protein performs the modification while the O-antigen side chain is being assembled (reviewed in Allison and Verma, 2000). Phylogenetic mapping of the GtrC proteins showed that there are at least 10 different ‘families’ of *gtr* operons in *S. enterica* genomes (Davies *et al*., 2013), and individual strains can encode for as many as four different *gtr* operons. It is thought that each family is responsible for a unique modification. Furthermore, the expression of many *gtr* operons is under the control of epigenetic phase variation (Broadbent *et al*., 2010a). Thus, the *gtr* repertoire of an isolate with four phase-varying *gtr* operons could result in up to 16 individual phenotypes within a population based just on the O-antigen composition.

Of the 10 GtrC families identified in Davies *et al*., the biochemical activity has been identified for only two, family 1 GtrC (serotype O1) and family 3 GtrC (serotype O12_2_). Specifically, the phage P22 family 1 gtrC operon results in the addition of a glucose residue via an α1– > 6 linkage to the galactose of the O-antigen subunit and the O1 serotype (Fukazawa and Hartman, 1964; Makela, 1973; Van der Byl and Kropinski, 2000). Recently, the factor O12_2_ modification was attributed to a *gtr* operon from family 3, which mediates addition of a glucose molecule to the galactose via an α1– > 4 linkage (Bogomolnaya *et al*., 2008). However, since the expression of both are under the control of phase variation, the associated factor O1 and O12_2_ modifications will not always be detectable (Broadbent *et al*., 2010a). To date, the activity of the remaining families of GtrC proteins has not been elucidated.

There is evidence that the Gtr-mediated modification helps prevent superinfection by altering the O-antigen (co)-receptor recognized by the phage, which provides a rationale for the presence of these genes on phage genomes. The P22 tailspike protein (TSP) contains endorhamnosidase activity to cleave the O-antigen and better gain access to the bacterial surface. This is blocked by α1–6 glucosylation of the galactose in the O-antigen subunit (O1 serotype), which is mediated by the P22 *gtr* operon. The rhamnosidase activity is not blocked by the chromosomally encoded family 3 *gtr* operon that results in α1–4 glucosylation (O12_2_ serotype). In contrast, phages 9NA and KB1 are able to cleave O-antigen containing the α1–6 glucosylation but not the α1–4 glucosylation, so there is some difference in the glycanase activity of different phages. P22 infection is also blocked by acetylation of the rhamnose (Wollin *et al*., 1987). More recently, the phase variable expression of the O12_2_ modification was demonstrated to directly affect the ability of phage SPC35 to infect *S. Typhimurium* (Kim and Ryu, 2012).

Many *gtr* operons have been retained in the genome of *Salmonella* serovars despite the degradation of the surrounding phage sequences (Davies *et al*., 2013). This indicates that these operons may provide some additional, direct benefit for the bacterium. Indeed, in *S. flexneri*, glucosylation by the products encoded in the *gtrV* operon increases virulence of the strain by compacting the O-antigen layer to allow the type 3 secretion system better access to epithelial cells (West *et al*., 2005). In *S. Typhimurium*, the family 3 *gtr-*mediated glucosylation of the O-antigen is associated with an increase in the persistence of the bacteria in the intestine (Bogomolnaya *et al*., 2008).

The *gtr* operon encoded by BTP1 is classified as a family 2 *gtr* operon (Davies *et al*., 2013); this family is associated with invasive *Salmonella* serovars, such as *S*. Typhi and *S*. Paratyphi A. D23580 was the first *S. Typhimurium* isolate in which a family 2 *gtr* operon was identified, which suggests that the modification performed by this operon may have contributed to the invasive capacity of this invasive non-typhoidal Salmonellae (iNTS) isolate. In this study we examined the functionality and significance of the prophage-encoded *gtr*^BTP1^ present in the iNTS strain D23580.

## Results

### The *gtr*^BTP^^1^ operon is required for full length of O-antigen in iNTS strains with BTP1 prophage

**Fig 1:**
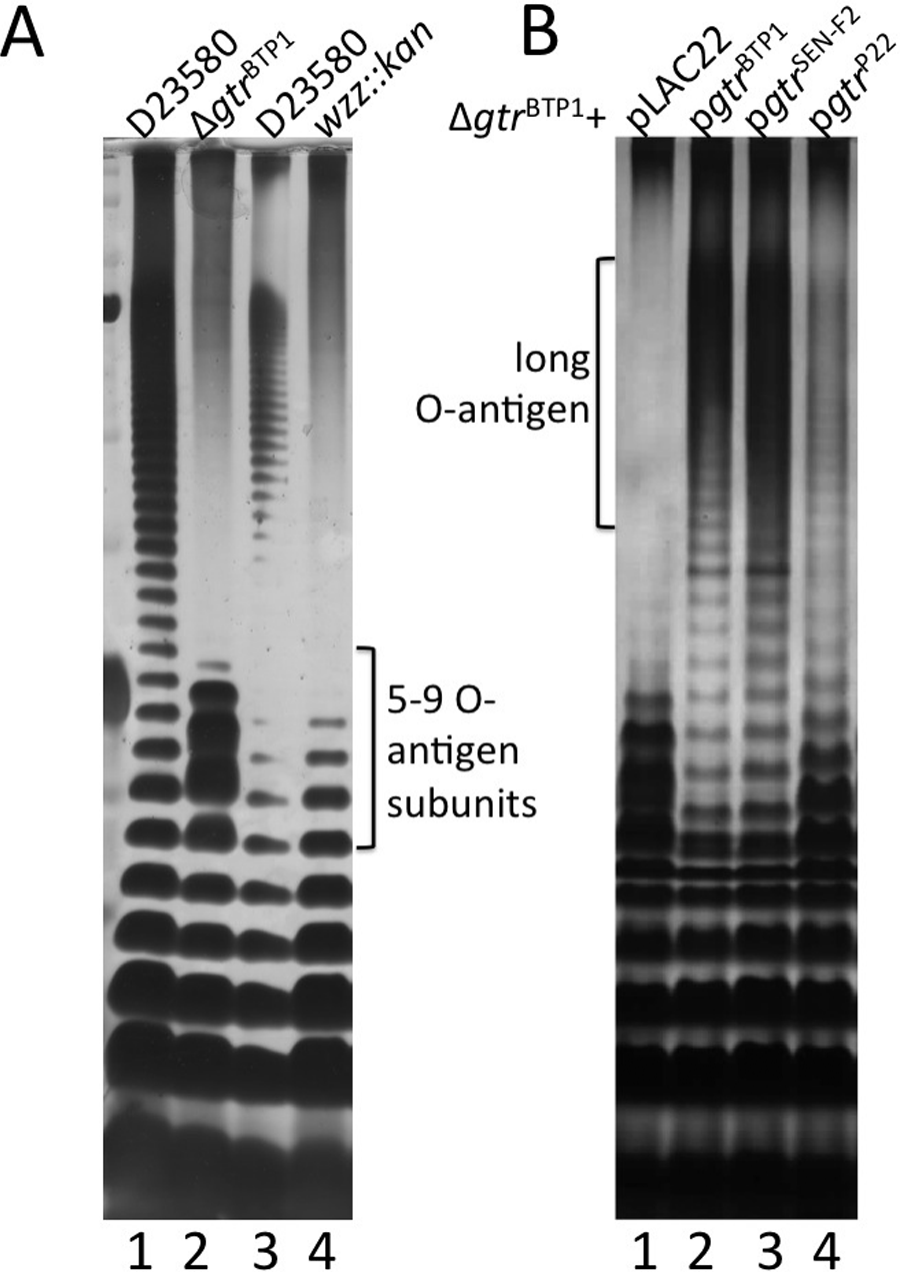
Deletion of the *gtr*^BTP^^1^ operon in *S.* *Typhimurium* D23580 affects O-antigen length. Crude LPS was run on a TSDS–PAGE gel and silver stained (see Experimental procedures section). Shown is LPS from stationary phase cultures of strain D23580 and its derivatives grown in LB; each rung represents a 1 unit difference in O-antigen length.A. The O-antigen phenotype of the Δ*gtr^BTP1^* mutant does not resemble a *wzz* mutant. Lanes are as follows: (1) D23580; (2) Δ*gtr^BTP1^*; (3) D23580; (4) *wzz*::kan.B. Family 2 *gtr* operons complement the short O-antigen phenotype seen in Δ*gtr^BTP1^*. Lanes are as follows: (1) Δ*gtr*^BTP1^ + pLAC22; (2) Δ*gtr*^BTP1^ + p*gtr*^BTP1^; (3) Δ*gtr*^BTP1^ + p*gtr*^SEN-F2^; (4) Δ*gtr*^BTP1^ + p*gtr*^P22^. SEN-F2 = *S.* Enteritidis family 2 *gtr* operon.

To assess the role of the family 2 *gtr*^BTP1^ operon (STMMW_03911–03921), these two genes were deleted and introduced into the native BTP1 prophage in *S. Typhimurium* D23580 (sMV189), resulting in Δ*gtr*^BTP1^ (sMV386). The LPS was analyzed on a sodium dodecyl sulfate–polyacrylamide gel electrophoresis (SDS–PAGE) gel to determine if a shift could be observed that would be indicative of addition of a sugar moiety (Davies *et al*., 2013). Unexpectedly, the O-antigen of this Δ*gtr*^BTP1^ strain was very short (Fig. 1A, lane 2). The O-antigen of the Δ*gtr*^BTP1^ strain differs from that of a mutant in the *wzz* gene, which is responsible for producing the long O-antigen chain length; this difference is especially evident in the range of 5–9 O-antigen subunits (Fig. 1A, lane 2 *vs.* 4). The Δ*gtr*^BTP1^ LPS phenotype also differed from a rough strain that lacks an O-antigen (data not shown). This indicates the *gtr*^BTP1^ mutation did not affect the core LPS and O-antigen biosynthesis pathways, and the reduced O-antigen length is due specifically to the absence of *gtr*^BTP1^. The short O-antigen phenotype was complemented by the cloned *gtr*^BTP1^ operon (sMV359) as well as by the family 2 *gtr* operon from *S.* Enteritidis PT4 (referred to as SEN-F2; sMV338); the GtrC^SEN-F2^ protein shares 77% identity with GtrC^BTP1^. However, complementation with the family 1 phage P22 *gtr* operon (sMV531) only partially restored the amount of long O-antigen (Fig. 1B).

### GtrC^BTP^^1^ does not require GtrA/B for activity

Comparative genome analysis showed that family 2 *gtr* operons differed from other *gtr* operons due to the presence of a truncation of the *gtrB* gene (Davies *et al*., 2013). Analysis of specifically the *gtr*^BTP1^ family 2 operon identified additional differences. First, *gtr*^BTP1^ has a complete *gtrB* gene deletion, unlike the C-terminal *gtrB* truncation found in other family 2 *gtr* operons (Davies *et al*., 2013). Second, *gtrA*^BTP1^ (referred to further as gtrA*) has a premature stop codon at nt 276 of the coding sequence, compared with one at nt 390 in the *S.* Typhi and *S*. Enteritidis family 2 *gtr* operons.

**Fig 2:**
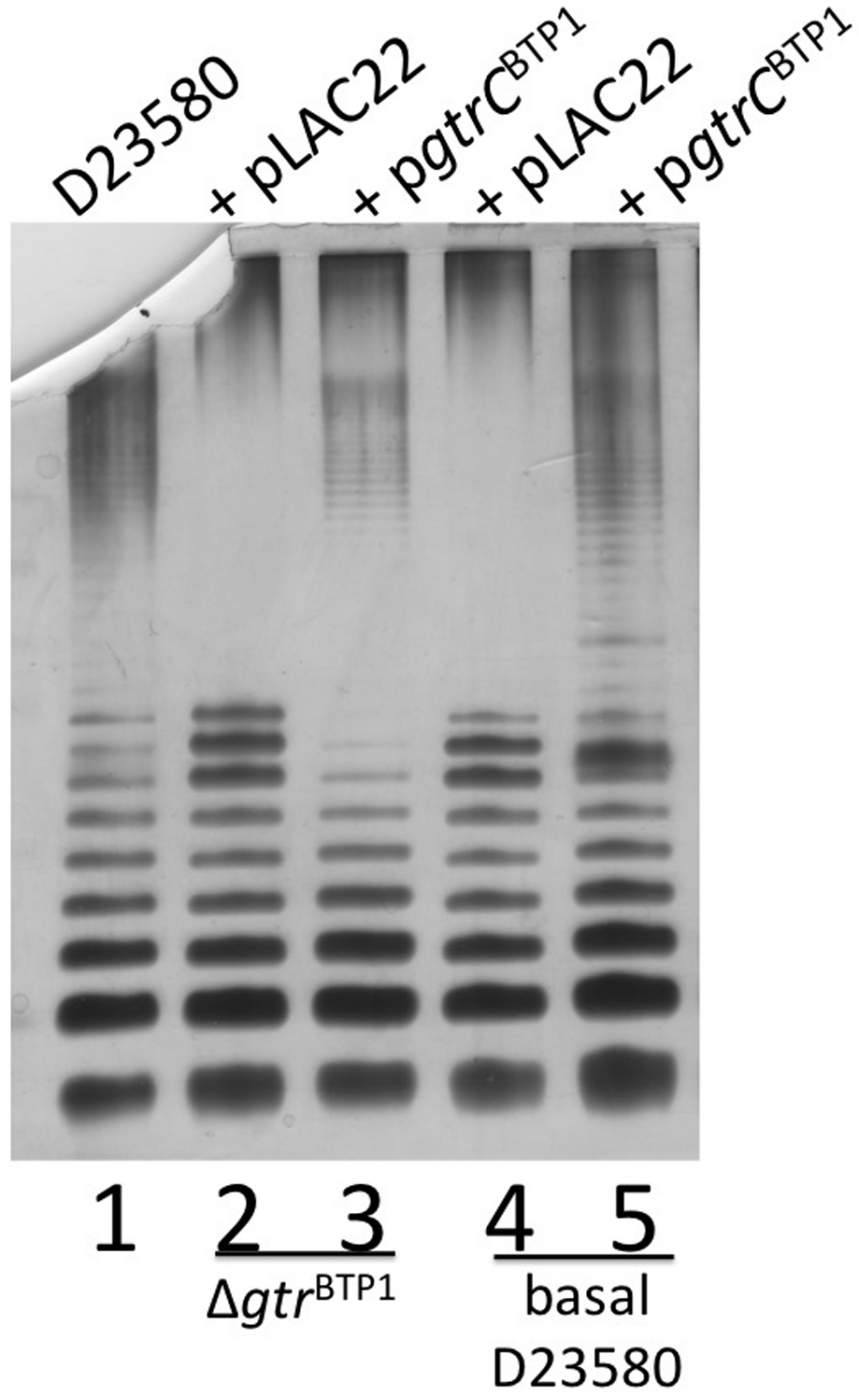
GtrA and GtrB are not required for biological activity of GtrC^BTP^^1^. LPS analysis details are as in legend for Fig. 1. Lanes are as follows: (1) D23580; (2) D23580 Δ*gtr*^BTP^^1^ + pLAC22; (3) D23580 Δ*gtr*^BTP^^1^ + p*gtrC*^BTP^^1^; (4) basal D23580 + pLAC22; (5) basal D23580 + p*gtrC*^BTP^^1^.

The mutations associated with the *gtr*^BTP1^ operon suggest that the GtrA/B proteins are no longer necessary for the activity of GtrC. Indeed, the Δ*gtr*^BTP1^ short O-antigen phenotype can be complemented by providing just GtrC^BTP1^ *in trans* (Fig. 2, lane 3). However, another possibility is that GtrA/B complementation occurs from one of the other chromosomal *gtr* operons. This is conceivable since the amino acid sequence identity of GtrA and GtrB between different *gtr* families is high, and the D23580 chromosome codes for family 3 and family 4 *gtr* operons in addition to *gtr*^BTP1^ (Davies *et al*., 2013). To address the necessity of GtrA/B for GtrC^BTP1^ functionality, a D23580 derivative with deletion of all three different *gtr* operons was constructed (‘basal’ D23580 strain) (sMV688). In this strain with an empty vector, the O-antigen is short (Fig. 2, lane 4). Expression of long O-antigen is restored by complementation with just GtrC^BTP1^ (Fig. 2, lane 5). This shows that GtrC^BTP1^-dependent O-antigen modification does not require any GtrA or GtrB proteins. Furthermore, this indicates a mechanistic difference between modification by family 2 GtrC proteins and GtrC proteins from families with conserved *gtrAB* genes.

### Identifying key features of the GtrC^BTP^^1^ protein

**Fig 3:**
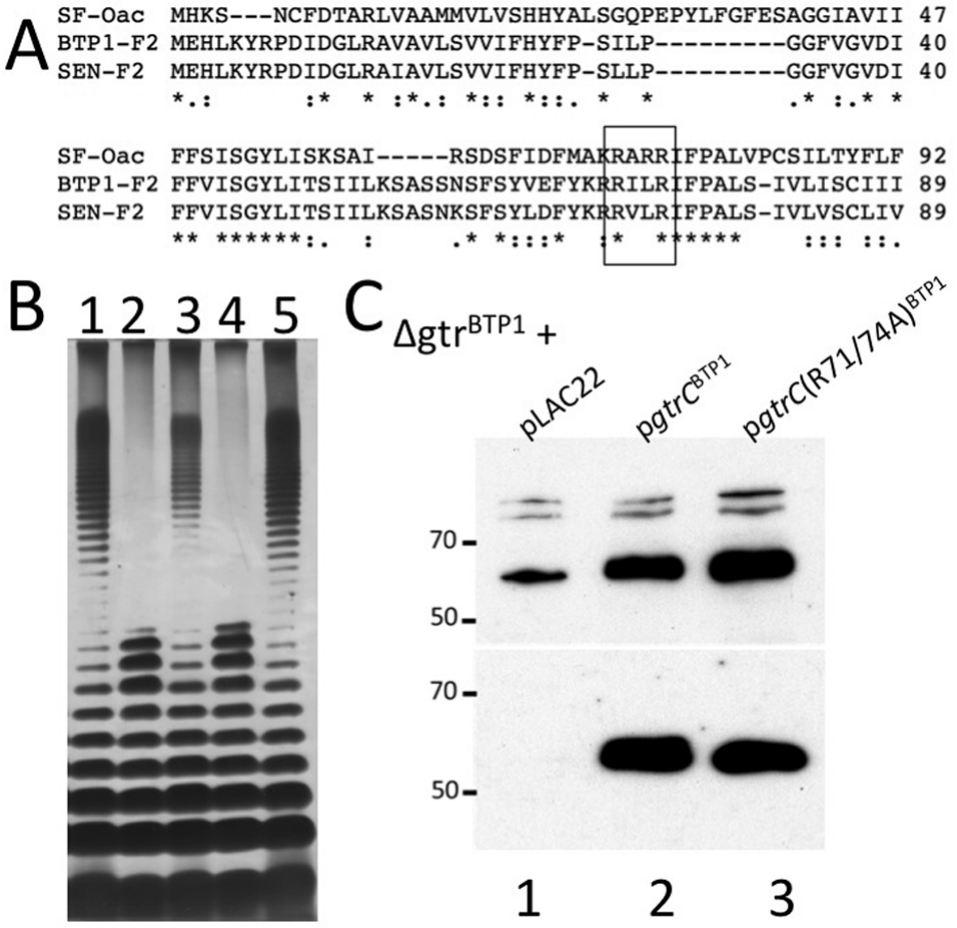
The RXXR motif is required for GtrC^BTP^^1^ biological activity.A. Family 2 GtrC proteins share homology with O-antigen acetyltransferase Oac from *S. flexneri* (SF). The RXXR motif found to be necessary for Oac activity is highlighted in the boxed region. Alignment was performed using TCOFFEE. SEN = *S.* Enteritidis; F2 = Family 2 GtrC.B. LPS analysis as in legend for Fig. 1. Lanes are as follows: (1) D23580; (2) Δ*gtr*^BTP1^ + pLAC22; (3) Δ*gtr*^BTP1^ + p*gtrC*^BTP1^; (4) Δ*gtr*^BTP1^ + p*gtrC*(R71/74A)^BTP1^; (5) D23580.C. Membrane localization of FLAG-tagged GtrC^BTP1^ and its R71A/R74A mutant derivative. Shown is a Western blot probed with anti-FLAG of WCL (top) and crude membrane extracts (bottom) made from D23580 Δ*gtr*^BTP1^ containing indicated vectors. Lanes: (1) pLAC22; (2) Δ*gtr*^BTP1^ + p*gtrC*^BTP1^; (3) Δ*gtr*^BTP1^ + p*gtrC*(R71/74A)^BTP1^.

Alignment of the family 2 GtrC proteins with members of GtrC family 1 or 3, which have documented glucosylation activity, shows the former have a 33 amino acid conserved region toward the N-terminus that is not shared with the GtrC proteins from the other families (data not shown). BLAST analysis showed that this region aligned with the N-terminus of the *S. Typhimurium* O-antigen acetyltransferase OafA, and also contains the RXXR motif that is required for Oac-dependent acetylation of the O-antigen in *S. flexneri* (Thanweer and Verma, 2012) (Fig. 3A). A site-directed mutant in the RXXR motif was generated to determine if this region was also important for GtrC^BTP1^ activity; the R71A/R74A mutation of the GtrC^BTP1^ protein (sMV648) failed to complement the short O-antigen phenotype of the Δ*gtr*^BTP1^ strain (Fig. 3B, lane 4). This mutant GtrC protein is still expressed and can be detected in the membrane at levels similar to wild-type GtrC (Fig. 3C). These data show that the RXXR motif is required for GtrC^BTP1^ activity, in the same way as it is required for Oac acetylation in *S. flexneri*.

**Fig 4:**
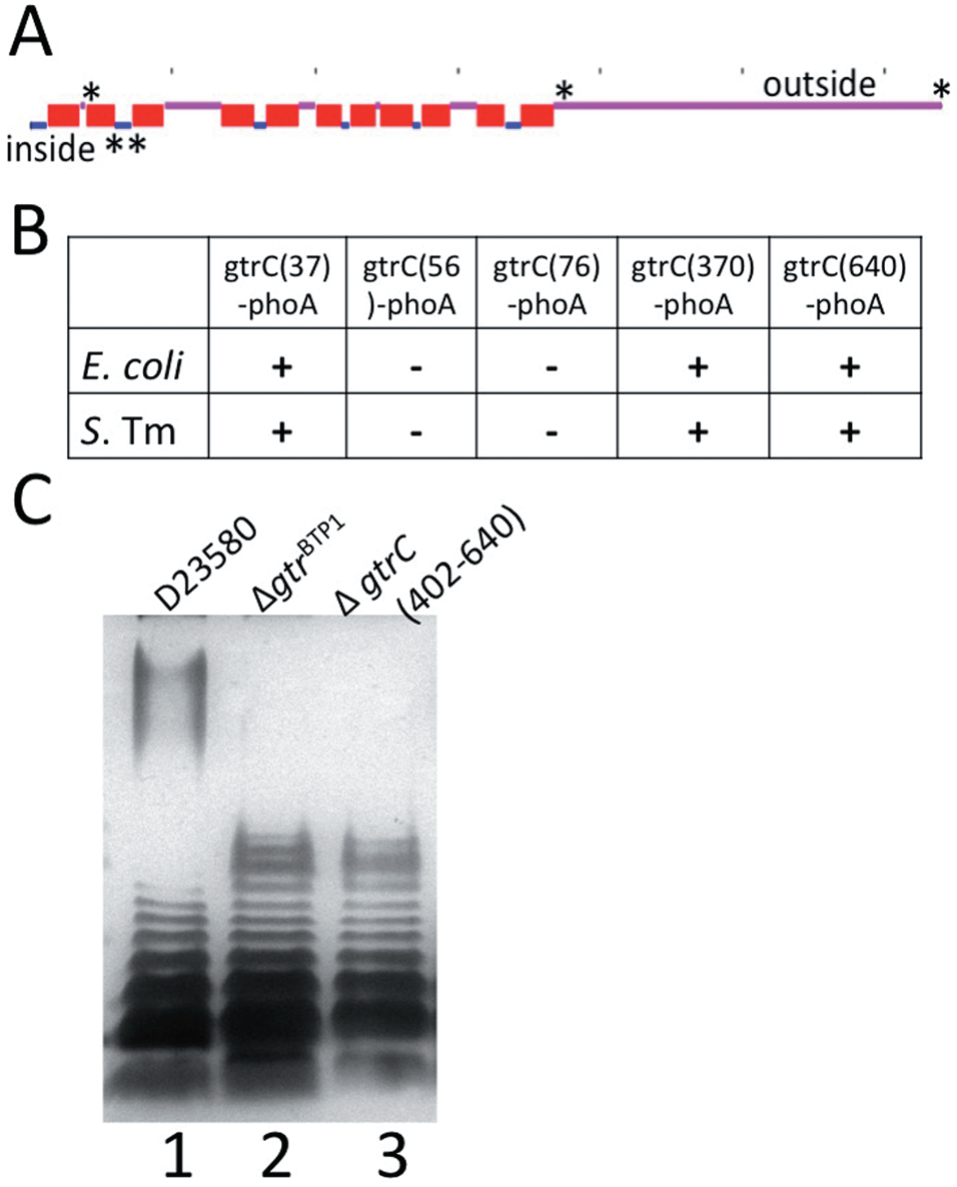
Membrane topology of GtrC^BTP^^1^ and requirement for the C-terminal periplasmic domain for activity.A. Locations of the GtrC^BTP1^–PhoA fusions along the TMHMM (Moller *et al*., 2001) topology prediction are given with an asterisk (*). Dashes along top indicate 100aa increments.B. Phenotype of GtrC^BTP1^–PhoA fusion in both *E. coli* and *S. Typhimurium* (*S*. Tm) strain 4/74. (−) indicates white color and (+) blue color. The location of the amino acid fusion is given in the vector name along the top row.C. The C-terminus of GtrC^BTP1^ is required for activity. LPS analysis as in the legend for Fig. 1. Lanes are as follows: (1) D23580; (2) Δ*gtr*^BTP1^; (3) *gtrC*(Δ402–640)^BTP1^.

GtrC^BTP1^ distinguishes itself from *S. flexneri* Oac, first, by being in the context of a (remnant) *gtr* operon. Second, topological analysis demonstrated that Oac has 10 transmembrane domains and a C-terminal cytoplasmic domain (Thanweer and Verma, 2012), whereas GtrC^BTP1^ is predicted to have 11 transmembrane domains and a 273 amino acid long C-terminus in the periplasm (Fig. 4A). To determine the localization of the RXXR motif and the C-terminal domain, GtrC^BTP1^ protein topology was analyzed with PhoA protein fusions. The PhoA enzyme is only active in the periplasm, so the location of loops between transmembrane domains can be determined by assessing the absence or presence of PhoA enzymatic activity in strains containing these fusions. Five different fusions were created (vectors pMV406-410) and PhoA activity was investigated in both *Escherichia coli* and *S. Typhimurium*. A fusion at amino acid 37, at the end of the first predicted transmembrane domain, was PhoA-positive, whereas fusions at amino acids 56 and 76 were PhoA-negative; the latter two amino acids flank the RXXR loop, indicating those essential amino acids are located in the cytoplasm (Fig. 4B). In agreement with the topology prediction, PhoA fusions at amino acids 370 (at the beginning of the predicted C-terminal periplasmic region) and 640 (at the C-terminal end) resulted in PhoA-positive colonies, indicating periplasmic localization. GtrC^BTP1^ activity furthermore requires at least the final 238 amino acids of the predicted 273 amino acid periplasmic C-terminus, as the O-antigen of a strain with *gtrC*^BTP1^ truncated at amino acid 402 (sMV548) resembles that of the Δ*gtr*^BTP1^ strain (sMV386) (Fig. 4C). These data are consistent with the long C-terminus of GtrC^BTP1^ being located in the periplasm and having an essential role for the biological function of the protein.

Taken together, the data described earlier suggest that the family 2 *gtr* operon encodes an O-antigen acetyltransferase. First, there is no requirement for GtrAB proteins that are necessary for glucose modification, and second, the RXXR motif is essential, similar to the role of this motif in *S. flexneri* O-antigen acetyltransferase Oac.

### *gtrC*^BTP^^1^ expression is not phase variable

Expression of most *gtr* operons across the *gtr* families, except family 4, is under the control of phase variation associated with a signature sequence in the regulatory region (Broadbent *et al*., 2010a; Davies *et al*., 2013). The sequence upstream of gtrA* in BTP1, however, contains a 365 nucleotide sequence that disrupts this signature sequence. To assess whether the insertion disrupts phase variation, a strain carrying a fusion of this region from *gtr*^BTP1^ to *lacZ* was analyzed. This strain gave rise to colonies with a uniform Lac+ (ON) phenotype (data not shown), reflecting a lack of phase variation.

**Fig 5:**
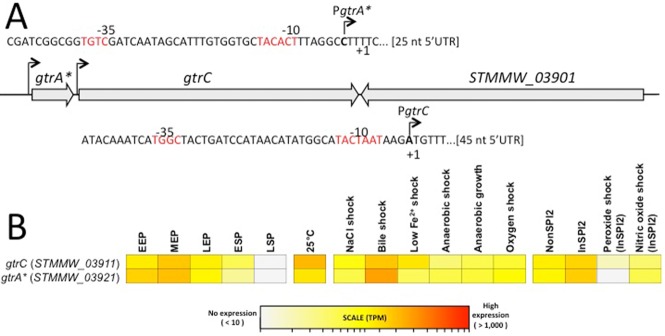
Genetic organisation and transcriptional profiling of the *gtr*^BTP^^1^ locus. The gtrA*, *gtrC* and *STMMW_03901* (TSP) genes are encoded within the phage BTP1 locus in *S. Typhimurium* strain D23580 (Kingsley *et al*., 2009).A. The locations of the transcription start sites (TSS) of *gtrC* (*STMMW_03911*) and gtrA* (*STMMW_3921*) are shown, and the predicted -10/-35 promoter motifs and the initiating nucleotide of both *gtr* transcripts are highlighted in bold. The nucleotide position of the *gtrC* (*STMMW_03911*) gene is 406441–408363 (negative strand), the gtrA* (*STMMW_3921*) gene is 408435–408710 (negative strand) and the *STMMW_03901* gene is 404416–406401 (positive strand) on the D23580 chromosome.B. Absolute gene expression of the *gtr*^BTP1^ locus. The heat map shows the levels of gene expression of the *gtr*^BTP1^ genes as TPM values, derived as described (Kroger *et al*., 2013).

Two transcriptional start sites (TSS) of *gtr*^BTP1^ were identified in strain D23580 by differential RNA-seq analysis. One TSS was upstream of gtrA*, and the second upstream of *gtrC*^BTP1^ (Fig. 5A). The former is the same TSS previously identified for the *gtr^LT2_I^* operon (STM0559-7) in *S. Typhimurium* strain LT2 that is phase variable (Broadbent *et al*., 2010b). However, as shown earlier, in strain D23580, transcription from this promoter region is not controlled by phase variation. The second TSS is in a region created by the *gtrB* deletion in D23580 and presumably would not be present in other *gtr* operons. Transcriptomic data from RNA-seq experiments (Fig. 5B) indicate that both gtrA* and *gtrC*^BTP1^ are usually expressed, and therefore a degree of O-antigen modification is likely to occur, under a wide range of growth conditions. These ranged from standard laboratory growth conditions to those designed to mimic *in vivo* infections (Kroger *et al*., 2013; see Experimental procedures section for further details). Future work is needed to determine the relative contribution of the two promoters for *gtrC*^BTP1^ expression.

### The Δ*gtr*^BTP^^1^ effect on O-antigen length requires the BTP1 phage tailspike protein

Analysis of the sequence adjacent to the *gtr*^BTP1^ operon identified gene STMMW_03901, which is convergently transcribed (see Fig. 5A). STMMW_03901 encodes for a TSP with 70% identity to that of the well-studied *Salmonella* phage P22 protein. The phage P22 TSP has endorhamnosidase activity that allows cleavage of the O-antigen. Specific amino acids important for this enzymatic activity of P22 TSP (namely, Glu 359, Asp 390 and Asp 392) are present in BTP1 TSP (Baxa *et al*., 1996; Steinbacher *et al*., 1996). Thus, the shorter O-antigen of the Δ*gtr*^BTP1^ strain could be due to BTP1 TSP-dependent cleavage of full length O-antigen.

**Fig 6:**
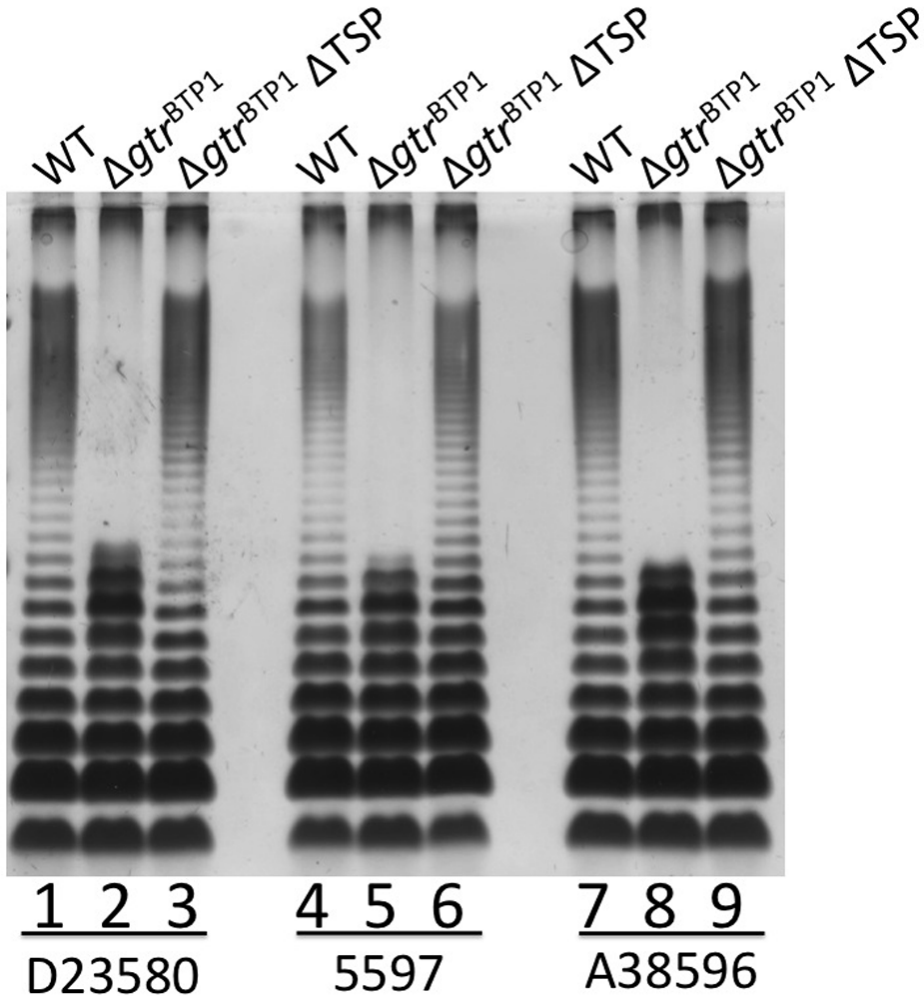
O-antigen length in iNTS strains with the BTP1 prophage is defined by a combination of the TSP rhamnosidase and GtrC^BTP^^1^ activity. LPS analysis as in legend for Fig. 1. Lanes are as follows: (1) D23580; (2) D23580 Δ*gtr^BTP^^1^*; (3) D23580 Δ*gtr^BTP^^1^*ΔTSP; (4) iNTS strain 5597; (5) 5597 Δ*gtr^BTP^^1^*; (6) 5597 Δ*gtr^BTP^^1^*ΔTSP; (7) iNTS strain A38596; (8) A38596 Δ*gtr^BTP^^1^*; (9) A38596 Δ*gtr^BTP^^1^*ΔTSP. WT = wild-type strain.

This hypothesis was tested by deleting the TSP gene in the D23580 Δ*gtr*^BTP1^ mutant (Fig. 6). When the Δ*gtr*^BTP1^ mutation is combined with the TSP deletion, the same full length O-antigen is seen as in the wild-type strain (Fig. 6, lane 3). This correlation between O-antigen length, depending on the presence of *gtrC*^BTP1^ and the TSP with its associated rhamnosidase activity, held true for two other iNTS strains (5597 and A38596) that are phylogenetic relatives of D23580 and harbor the BTP1 prophage (Kingsley *et al*., 2009; Okoro *et al*., 2012) (Fig. 6, lanes 4–9). These data lead us to conclude that in wild-type D23580, GtrC^BTP1^ acts to protect the O-antigen from cleavage by the phage BTP1 tailspike-encoded rhamnosidase activity.

### Significance of GtrC^BTP^^1^ in phage–bacteria interactions

The findings described earlier are consistent with a model in which prophage-encoded GtrC^BTP1^ mediates O-antigen modification, possibly via acetylation. Modification of a receptor is a common phage strategy to protect a lysogen from superinfection by phages that use the same (co)-receptor, in this case the O-antigen. Phage P22 is known to bind O-antigen as a receptor via its TSP and the BTP1 TSP sequence is homologous to that of P22 (Steinbacher *et al*., 1997), suggesting that BTP1 may also use the O-antigen as a (co)-receptor. Furthermore, our data indicate that unmodified, full-length O-antigen on the cell surface can be cleaved by the BTP1 phage TSP similar to P22 (Steinbacher *et al*., 1997), which can facilitate phage penetration.

**Fig 7:**
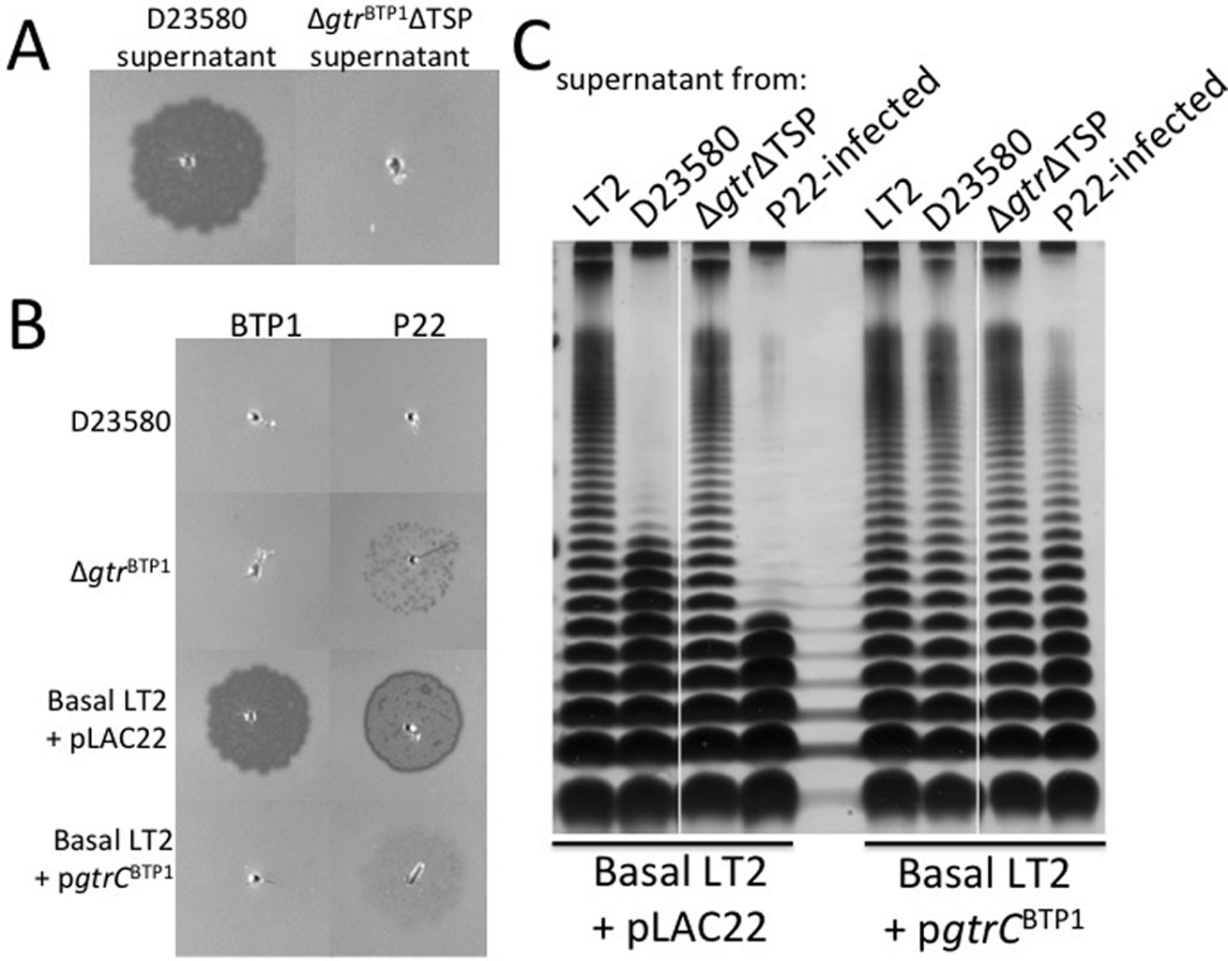
GtrC^BTP^^1^ O-antigen modification prevents infection by both P22 and BTP1 and inhibits cleavage of the O-antigen by the BTP1 phage.A. Supernatant from D23580 cultures, containing BTP1 phage, or the Δ*gtr*ΔTSP mutant were spotted on lawns of basal LT2 + pLAC22.B. Supernatant from cultures contacting BTP1 or P22 phage were spotted at equal titer (about 3.5 × 10^5^ pfu ml^−1^) on lawns of wild-type D23580, the Δ*gtr*^BTP1^ mutant, basal LT2 + pLAC22 or basal LT2 + p*gtrC*^BTP1^.C. Cultures of basal LT2 containing either empty vector or p*gtrC*^BTP1^ were incubated with supernatant of cultures from strains (as indicated along top of gel) to investigate O-antigen cleavage from the surface. LPS analysis performed as described in Fig. 1.

In this model, the short O-antigen of the Δ*gtr*^BTP1^ strain is a result of BTP1 phage-dependent O-antigen cleavage and, therefore, free phage BTP1 particles should be present in cultures of strain D23580. Indeed, plaques are observed upon spotting supernatant of a culture of strain D23580 on a lawn of ‘basal’ LT2, which is a mutant that lacks both native *gtr* operons (Fig. 7A). No plaques were observed with the supernatant of a D23580 mutant that has the BTP1 TSP gene deleted from the prophage, indicating these plaques are derived from BTP1 phage and not from other prophage present in this strain (Fig. 7A) (Kingsley *et al*., 2009). Furthermore, culture supernatants from the D23580-related iNTS strains A130 and A38596 also showed plaque formation, indicative of free BTP1 phage (data not shown).

To examine whether the GtrC^BTP1^ O-antigen modification affects phage susceptibility, spot titer tests were carried out with the BTP1- and P22-containing supernatants. Plaque formation by these phages was tested against the basal LT2 strain either with or without GtrC^BTP1^, and against wild-type D23580 and the Δ*gtr*^BTP1^ mutant. As expected, no plaque formation occurs on a D23580 lawn, consistent with an active phage repressor system. Interestingly, some P22 plaques did form on the Δ*gtr*^BTP1^ mutant, suggesting that P22 is a heteroimmune phage (right column, Fig. 7B). Importantly, GtrC^BTP1^ activity blocked infection by both phages BTP1 and P22 (Fig. 7B). Specifically, BTP1 and P22 formed plaques on the basal LT2 strain containing only empty vector (Fig. 7B). This plaque formation, however, is absent when spotting BTP1 phage on this strain containing p*gtrC*^BTP1^ (sMV816). Similarly, P22 infection of LT2 is strongly inhibited by p*gtrC*^BTP1^ (Fig. 7B). P22 also does not infect wild-type D23580 but can infect the Δ*gtr*^BTP1^ D23580 mutant. These data illustrate the protective nature of the modification of the O-antigen by GtrC^BTP1^.

Importantly, both the BTP1 and P22 phages mediated cleavage of the full length O-antigen present on basal LT2 (Fig. 7C, lanes 2 and 4). When the GtrC^BTP1^ protein was present, O-antigen was not cleaved from the surface of the bacteria and full length LPS was maintained (Fig. 7C, lanes 6 and 8). No O-antigen cleavage was observed with supernatant from the Δ*gtr*^BTP1^ΔTSP strain that lacks both *gtr*^BTP1^ and TSP (Fig. 7C, lanes 3 and 7). Thus, GtrC^BTP1^ activity protects the O-antigen from BTP1 TSP mediated O-antigen cleavage.

**Fig 8:**
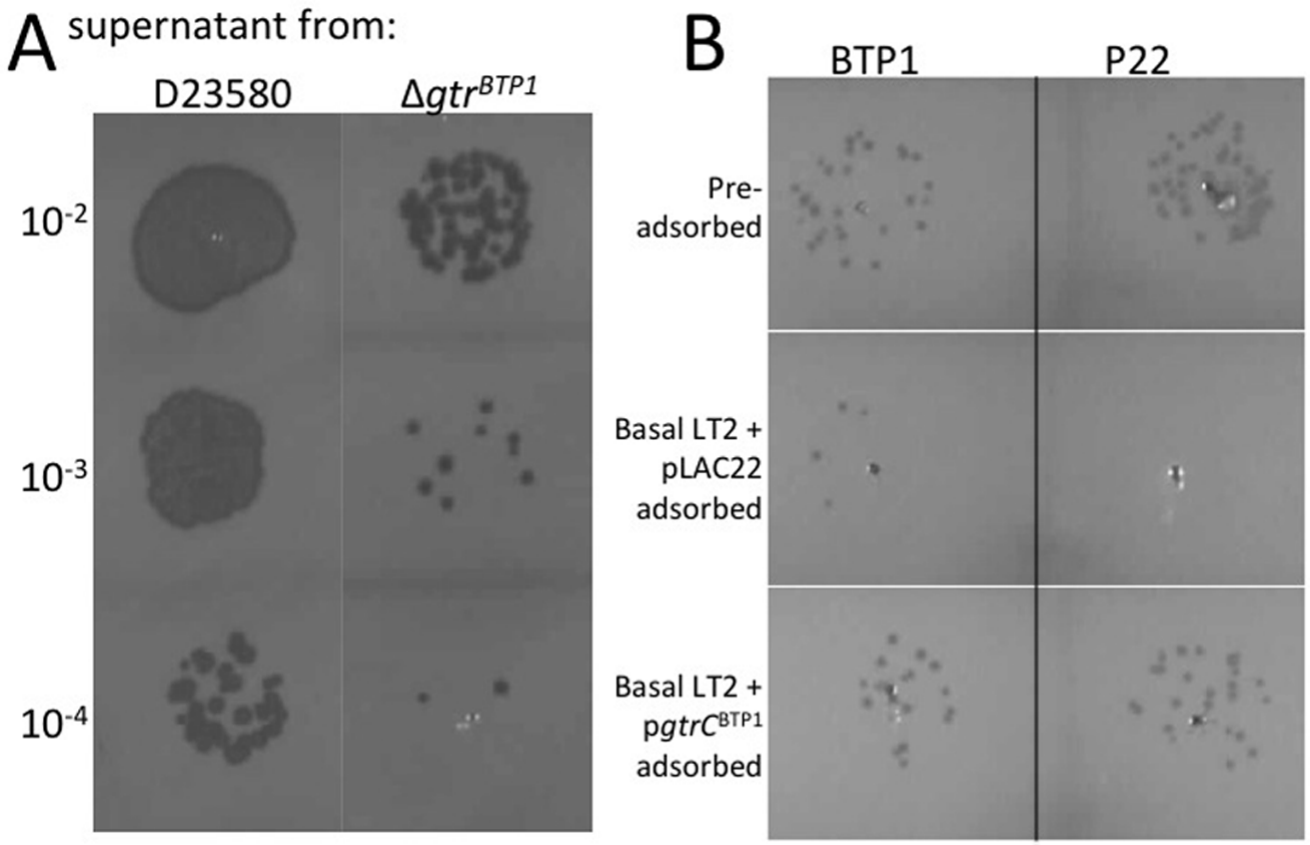
Modification by GtrC^BTP^^1^ prevents readsorption of phage.A. BTP1 titer is reduced in a strain with a deletion of *gtr^BTP1^*. Supernatants containing BTP1 phage, obtained from a D23580 culture or the Δ*gtr^BTP1^* mutant, were spotted on lawns of basal LT2. Dilutions from the original supernatant are given.B. BTP1 and P22 phage titers are affected by adsorption with strains having or lacking GtrC^BTP1^ O-antigen modification. BTP1 and P22 phage were incubated for 5 minutes with the indicated strains (top row shows phage titer pre-adsorption). Bacteria and phage bound to their surface were removed by pelleting and the free, unadsorbed phage numbers determined by spotting the supernatant on a lawn of basal LT2. The same dilution of phage for all samples is shown.

Another role of phage receptor modification could be the prevention of readsorption of phage released by lysogenic kin. To address whether GtrC^BTP1^-mediated O-antigen modification can prevent readsorption, phage titers from wild-type D23580 and the Δ*gtr*^BTP1^ mutant were compared (Fig 8A). Phage concentrations produced by overnight cultures of D23580 were 5.8 × 10^7^ pfu/ml, but the Δ*gtr*^BTP1^ mutant only produced 1.4 × 10^6^ pful/ml. The lower titers in the absence of GtrC^BTP1^ could be due to phage particles rebinding to the surface of the bacteria that express an un-modified O-antigen. To further test adsorption directly, BTP1 phage-containing supernatant was incubated with the LT2 strain that lacks O-antigen modification, and with this strain expressing GtrC^BTP1^ (Fig. 8B). Adsorption with the unmodified strain resulted in a greater decrease in titer than adsorption with the strain expressing modified O-antigen. This is consistent with the conclusion that modification can reduce readsorption of phage. Less adsorption to the LT2 strain with modified O-antigen was also observed with P22 phage, consistent with the lower level of infection observed on strains with GtrC^BTP1^-mediated O-antigen modification (Fig. 7). Together, our data support a model in which BTP1 phage-encoded GtrC modifies the BTP1 O-antigen (co)-receptor to reduce superinfection by heteroimmune phages that use the same O-antigen moiety as BTP1 as co-receptor, and to limit readsorption by kin.

## Discussion

The BTP1 prophage is one of the distinguishing genetic features of the iNTS *S. Typhimurium* strain D23580 (Kingsley *et al*., 2009). Previously, a *Salmonella*-wide genome analysis for the occurrence of O-antigen modifying *gtr* operons determined that this prophage encoded an uncharacterized *gtr* operon (Davies *et al*., 2013). Here we analyzed the operon and its significance for this strain. This *gtr* operon has one full-length protein: the GtrC^BTP1^ protein. We determined that GtrC^BTP1^ has hallmarks consistent with acetyltransferase activity. Expression of GtrC^BTP1^ occurs under a wide range of growth conditions that are relevant to infection, and, unlike many other *gtr* operons, expression of *gtr^BTP1^* is not subject to phase variation. We show that the GtrC^BTP1^-mediated modification plays an important role in the bacterium–phage interaction by protecting the bacterium from infection by both BTP1 and the related P22 phage.

Previously characterized *gtrABC* operons mediate the glucosylation of the O-antigen, but have different receiving moieties and linkages (Allison and Verma, 2000). In contrast, our data suggest that the *gtr^BTP1^* locus mediates acetylation of the O-antigen, and we propose that GtrC^BTP1^ is the protein responsible for the acetylation of the rhamnose residue in the O-2 and O-3 positions that was recently identified in biochemical analysis of the O-antigen of strain D23580 (Micoli *et al*., 2014). First, GtrC^BTP1^ contains an acetyltransferase domain from the acyl_transf_3 superfamily within its first 360 amino acids. Second, we showed that a RXXR protein motif that is essential for the Oac acetyltransferase activity in *S. flexneri* (Thanweer and Verma, 2012) is present in GtrC^BTP1^ and also is essential for its activity (Fig. 3A and B). GtrA and GtrB have roles in transferring a glucose moiety across the inner membrane; the absence of *gtrB* and truncation of *gtrA*, and the fact they are not required for this modification (Fig. 2), provides further evidence that the activity of GtrC^BTP1^ does not involve glucosylation.

All the data are consistent with the conclusion that the family 2 GtrC^BTP1^ mediates acetylation of the rhamnose in the O-antigen. Based on high amino acid identity among the GtrC proteins of this family (Davies *et al*., 2013) and the ability of the *S.* Enteritidis family 2 *gtr* operon to complement the Δ*gtr*^BTP1^ short O-antigen phenotype, it is highly likely that all family 2 GtrC proteins have acetyltransferase activity. In support of this conclusion, analysis of the O-antigen of defined strains of *S*. Typhi confirmed that acetylation of the rhamnose requires the *S.* Typhi family 2 *gtr* operon (manuscript in preparation). This *S*. Typhi GtrC has 77% identity with GtrC^BTP1^ and 99% identity with the *S.* Enteritidis family 2 GtrC protein, which complements the short O-antigen phenotype of our Δ*gtr*^BTP1^ mutant. Thus there is strong support for the conclusion that all family 2 GtrC proteins carry out acetylation of the rhamnose in the O-antigen of these strains.

GtrC^BTP1^ and other O-antigen acetyltransferases, such as Oac in *S. flexneri* and OafA in *S. Typhimurium* (Slauch *et al*., 1996; Thanweer *et al*., 2008; Thanweer and Verma, 2012), share some predicted structural features. As noted earlier, all three acetyltransferase proteins contain the RXXR motif, which is necessary for the O-antigen modification by Oac and GtrC^BTP1^. A cytoplasmic location of this motif has been experimentally confirmed for Oac (Thanweer *et al*., 2008) and GtrC^BTP1^ (Fig. 4), and is predicted for OafA (Thanweer and Verma, 2012). The three acetyltransferases are also membrane proteins with multiple membrane spanning regions, ranging from 9 to 11 transmembrane domains. One of the significant differences between GtrC^BTP1^, OafA and Oac is the length and location of the C-terminal soluble domain. GtrC^BTP1^ and OafA contain a large (270 aa for GtrC^BTP1^, predicted 255 aa for OafA) periplasmic tail that is necessary for activity (Fig. 4C and Hauser *et al*., 2011). In contrast, Oac has a relatively short (22 aa), cytoplasmically located C-terminal end. The role and relevance of the location of the C-termini remain to be elucidated, but it suggests that *Salmonella* GtrC acetyltransferase enzymes have an additional activity or different mechanisms of activity than Oac from *S. flexneri*.

For these O-antigen acetyltranferases, it is unclear from where the acetyl moiety is derived, and where or when the moiety is transferred to the growing O-antigen chain. Given the requirement for both a cytoplasmic and a periplasmic domain, and the presence of multiple transmembrane domains, one possibility is that family 2 GtrC proteins function as a channel for the acetyl moiety in addition to carrying out the enzymatic transfer reaction. In this model, the RXXR cytoplasmic loop might be involved in binding the acetyl group for transfer to the periplasm. Alternatively, modification of the O-antigen subunit could occur in the cytoplasm as has been proposed for OafA (Slauch *et al*., 1996). The C-terminal domain may mediate necessary protein–protein interactions, potentially with the O-antigen assembly complex. Further work on the structure/function relationship of Gtr proteins is needed to elucidate these and many other aspects of *gtr*-mediated O-antigen modification.

Acetylation of the O-antigen, and specifically of the rhamnose moiety, has been previously described in context of lysogenic conversion by phages A3 and A4 (Wollin *et al*., 1987). This modification inhibited P22 adsorption and it was inferred that TSP-mediated O-antigen cleavage was inhibited (Wollin *et al*., 1987). Similarly, our data indicate that P22 and BTP1 phage-mediated infection is inhibited by GtrC^BTP1^ activity, likely because the acetyl modification blocks the TSP endorhamnosidase activity. This was evident from the short O-antigen phenotype in the absence of GtrC^BTP1^, and the dependency on the TSP for this phenotype. Effective protection against phage infection may also be facilitated by a high level of acetylation, with an estimated 80% of O-antigen subunits containing the modification in D23580 (Micoli *et al*., 2014). There are two contributing factors to this high degree of modification. First, GtrC^BTP1^ expression was not subject to phase variation, and thus all cells in a population will have modified O-antigen. This is in contrast to *gtr^P22^*-mediated glucosylation, for which the mixed population generated by phase variation was directly shown to affect the dynamics of interactions of a population of cells with phage (Kim and Ryu, 2012). Second, the fact that gtrA*C appears to be expressed in a wide range of growth conditions suggests that the O-antigen will usually be in the modified state (Fig. 5B). Thus, a benefit of the BTP1 prophage for the bacterium is that it confers effective protection on the entire bacterial population against infection by BTP1 and both homo- and heteroimmune phages that use the same O-antigen moiety as co-receptor.

There are several aspects of the BTP1 phage that could potentially contribute to the virulence of iNTS BTP1 lysogens. One is the high titer of BTP1 prophage (an average of 7 × 10^7^ pfu ml^−1^) we found in overnight cultures, even in the absence of phage-inducing conditions. Phage-associated lysis could produce bacterial debris, and this, in turn, could affect the development of the immune response to the iNTS infection. Additionally, the BTP1-encoded family 2 *gtr*^BTP1^ operon is not under the control of phase variation, whereas other family 2 operons are phase variable, including those from *S.* Enteritidis and *S.* Typhi and the majority of *gtr* operons (Broadbent *et al*., 2010a). Thus, D23580 does not have the option for phase-variable immune evasion from antibodies developed to the acetylated O-antigen, which could be significant. However, whether acetyl modification affects the course of infection has yet to be determined. The family 2 *gtr* operons are associated with many invasive serovars of *S. enterica*, and it will be important to determine whether, as we propose, O-antigen acetylation contributes to the typical course of infection, including systemic disease, as reported for the iNTS isolates.

## Experimental procedures

### Bacterial strains and culture conditions

Strains are listed in Table 1. Strains were maintained in either Luria broth or on Luria–Bertani (LB) plates at 37°C. Strains harboring temperature-sensitive vectors were grown at 30°C. For selection of mutants or maintenance of vectors, the following antibiotic concentrations were used: tetracycline (15 μg ml^−1^), ampicillin (100 μg ml^−1^), chloramphenicol (34 μg ml^−1^ for vectors or 8 μg ml^−1^ for chromosomal inserts) or kanamycin (30 μg ml^−1^).

**Table 1:** Strains used in this study

Strain name in text	Strain number	Relevant genotype	Plasmid	Source
*Escherichia coli* isolates				
*E. coli*–*gtrC*(37)^BTP1^–phoA	MV1557	NEB5a	pMV406	This study
*E. coli*–*gtrC*(56)^BTP1^–phoA	MV1558	NEB5a	pMV407	This study
*E. coli*–*gtrC*(76)^BTP1^–phoA	MV1559	NEB5a	pMV408	This study
*E. coli*–*gtrC*(370)^BTP1^–phoA	MV1560	NEB5a	pMV409	This study
*E. coli*–*gtrC*(640)^BTP1^–phoA	MV1561	NEB5a	pMV410	This study
*Salmonella* Typhimurium isolates				
D23580	sMV189	Wild-type		Kingsley *et al*., 2009
*gtrA^BTP1^*–*lacZ*	sMV523	sMV77att::pMV349		This study
Δ*gtr*^BTP1^	sMV386	D23580 Δ*gtr*^BTP1^ (STMMW_03921-STMMW_03911)		This study
Δ*wzz*	sMV664	D23580 *wzz*::kan (STMMW_21101)		This study
Δ*gtr*^BTP1^ + pLAC22	sMV610	D23580 Δ*gtr*^BTP1^	pLAC22	This study
Δ*gtr*^BTP1^ + p*gtr*^BTP^	sMV569	D23580 Δ*gtr*^BTP1^	pMV359	This study
Δ*gtr*^BTP1^ + p*gtr*^SEN-F2^	sMV571	D23580 Δ*gtr*^BTP1^	pMV338	This study
Δ*gtr*^BTP1^ + p*gtr*^P22^	sMV531	D23580 Δ*gtr*^BTP1^	pMV333	This study
Δ*gtr*^BTP1^ ΔTSP	sMV800	D23580 Δ*gtr*^BTP1^ ΔTSP		This study
Δ*gtr*^BTP1^ + p*gtr*C^BTP1^	sMV647	D23580 Δ*gtr*^BTP1^	pMV390	This study
Δ*gtr*^BTP1^ + p*gtr*C (R71/74A)^BTP1^	sMV648	D23580 Δ*gtr*^BTP1^	pMV392	This study
*gtr*C(Δ402–640)^BTP1^	sMV548	D23580 *gtrC*(402–640)^BTP1^::kan		This study
*S*. Tm-*gtrC*(37)^BTP1^–phoA	sMV697	*S. Typhimurium* 4/74	pMV406	This study
*S*. Tm-*gtrC*(56)^BTP1^–*phoA*	sMV698	*S. Typhimurium* 4/74	pMV407	This study
*S*. Tm-*gtrC*(76)^BTP1^–*phoA*	sMV699	*S. Typhimurium* 4/74	pMV408	This study
*S*. Tm-*gtrC*(370)^BTP1^–*phoA*	sMV700	*S. Typhimurium* 4/74	pMV409	This study
*S*. Tm-*gtrC*(640)^BTP1^–*phoA*	sMV701	*S. Typhimurium* 4/74	pMV410	This study
Basal D23580	sMV688	D23580 Δ*oafA*; Δ*gtr^BTP1^*; Δ*gtr*^Fam3^; Δ*gtr^Fam4^*		This study
Basal D23580 + pLAC22	sMV691	D23580 Δ*oafA*; Δ*gtr^BTP1^*; Δ*gtr*^Fam3^; Δ*gtr^Fam4^*	pLAC22	This study
Basal D2350 + p*gtr*C^BTP1^	sMV689	D23580 Δ*oafA*; Δ*gtr^BTP1^*; Δ*gtr*^Fam3^; Δ*gtr^Fam4^*	pMV390	This study
Basal LT2 + pLAC22	sMV815	LT2 Δ*oafA*; *gtr*^Fam3::^tet; Δ*gtr^Fam4^*::kan	pMVLAC22	This study
Basal LT2 + p*gtr*^BTP1^	sMV816	LT2 Δ*oafA*; *gtr*^Fam3::^tet; Δ*gtr^Fam4^*::kan	pMV390	This study
5597	sMV762	iNTS, lineage I		Kingsley *et al*., 2009
5597Δ*gtr^BTP1^*	sMV796	5597 *gtr^BTP1^*::tet		This study
5597Δ*gtr^BTP1^*ΔTSP	sMV801	5597 *gtr^BTP1^*::tet;TSP::kan		This study
A38596	sMV765	iNTS, lineage II		Kingsley *et al*., 2009
A38596Δ*gtr^BTP1^*	sMV792	A38596 A38596 *gtr^BTP1^*::tet		This study
A38596Δ*gtr^BTP1^*ΔTSP	sMV802	A38596 *gtr^BTP1^*::tet; TSP::kan		This study
A130	sMV761	iNTS, lineage I		Kingsley *et al*., 2009

### Molecular biology techniques

Standard molecular biology techniques were used (Sambrook *et al*., 1987). DNA was obtained using either genomic DNA isolated as described (Ausubel *et al*., 1989) or Qiagen miniprep kit for plasmids. PCRs were performed using either Merck's KOD Hot Start (Billerica, MA, USA) or Promega's GoTaq Flexi polymerases (Madison, WI, USA). Restriction enzymes and T4 ligase for cloning were purchased from NEB (Ipswich, MA, USA). All vectors and plasmids generated for this study are listed in Table 2. The site-directed mutagenesis was performed on constructs in the pLAC22 vector (Warren *et al*., 2000) with primers oMV954 (GAATTTTACAAGAGA**GCA**ATACTA**GCA**ATATTCCCAGCT) and oMV955 (AGCTGGGAATAT**TGC**TAGTAT**TGC**TCTCTTGTAAAATTC) using Agilent's QuikChange Lightning kit (Santa Clara, CA, USA). Bold regions indicate codons changed to introduce the mutations.

**Table 2:** Plasmids used in this study

	Parent plasmid	Description; gene cloned with oligonucleotides used	Source
pCP20-TcR	pCP20	TcR cassette inserted into SmaI site of pCP20 [oMV466; oMV467]^a^	This study
pMV349	pMV243	BTP1 regulatory region–*lacZ* fusion [oMV413; oMV875]	This study
pMV333	pLAC22	*gtrABC*(P22*)* [oMV776; oMV778]	Broadbent *et al*., 2010a
pMV338	pLAC22	*gtrABC*(*S.* Enteritidis family 2) [oMV776; oMV780]	This study
pMV359	pLAC22	*gtrABC*(BTP1) [oMV897; oMV898]	This study
pMV390	pLAC22	BTP1 GtrC-FLAG [oMV898; oMV958/959]	This study
pMV392	pLAC22	BTP1 GtrC (R54A/R57A)-FLAG; [oMV954; oMV955]	This study
pMV406	pSK4158	*gtrC*(BTP1)–*phoA* fusion at amino acid 37 [oMV993; oMV1003]	This study
pMV407	pSK4158	*gtrC*(BTP1)–*phoA* fusion at amino acid 56 [oMV994; oMV1003]	This study
pMV408	pSK4158	*gtrC* (BTP1)*–phoA* fusion at amino acid 76 [oMV995; oMV1003]	This study
pMV409	pSK4158	*gtrC* (BTP1)*–phoA* fusion at amino acid 370 [oMV996; oMV1003]	This study
pMV410	pSK4158	*gtrC* (BTP1)–*phoA* fusion at amino acid 640 [oMV997; oMV1003]	This study

a Oligos are given in Supplemental Table S1.

### Constructon of *lacZ* reporter fusions and detection of LacZ activity

The CRIM system was used to insert *gtr* promoter–*lacZ* fusions into the *attB* site of *S. Typhimurium* LT2 (Haldimann and Wanner, 2001) using vector pMV243 (Broadbent *et al*., 2010a). Strains containing inserts were streaked on minimal M9 media agar plates containing 40 μg ml^−1^ X-gal (5-bromo-4-chloro-3-indolyl-ß-d-galactoside) (Melford; Ipswich, Suffolk, UK).

### Generation of mutant strains

Allelic replacement was used to introduce mutations to the chromosome (Datsenko and Wanner, 2000). Antibiotic resistance cassettes were obtained from Tn10 for Tc-resistance (TcR) and pKD13 for KmR. To generate multiple mutations in a single strain, these resistance cassettes were occasionally removed (as indicated in Table 1) before the next mutation was introduced. Since the pKD13 KmR cassette is flanked by flippase recognition target (FRT) sites, the introduction of pCP20 (Cherepanov and Wackernagel, 1995) or its pCP20-TcR derivative allowed for the expression of Flp recombinase to produce unmarked mutant strains. When the Tn10 TcR cassette was used, lambda-red recombination was used to introduce an oligonucleotide homologous to the region surrounding the TcR cassette. This combined with counter-selection (Bochner *et al*., 1980) produced scarless mutations of the targeted genes. For example, to obtain strain Δ*gtr*^BTP1^ (sMV386), which contains a deletion of the BTP1 prophage-encoded gtrA* and the *gtrC* genes in D23580, the sequence from 101 nt upstream of the start codon of the pseudogene STMMW_03921 (gtrA*) to 15 nt downstream of the stop codon of STMMW_03911 (*gtrC^BTP1^*) was first replaced with a tetracycline cassette (amplified using oMV754/oMV755) (Table S1). This sequence was, in turn, replaced with oMV758 using the counter-selection method described. These genes are located on the phage genome between the phage TSP (TSP gene, STMMW_03901) and the phage–host genome junction.

### LPS extraction and visualization

Crude LPS extracts were prepared as described (Davies *et al*., 2013). After running the samples on TSDS–PAGE (Tricine-SDS–PAGE) gels, two methods were used for visualization: either silver stain as described previously (Davies *et al*., 2013) or Western blot. For Western blots, the samples were transferred to polyvinylidene fluoride (PVDF) and blocked with phosphate buffered saline with 0.1% Tween-20 (PBS-T) and 5% milk. The O4 serum from Statens Serum Institute (Denmark) was used as primary antibody followed by goat anti-rabbit IgG-HRP from Sigma (A0545). Millipore's Luminato Western HRP substrate was used for detection.

### Membrane preparation and FLAG visualization

Vectors encoding for FLAG-tagged versions of the GtrC^BTP1^ protein were expressed in Δ*gtr^BTP1^*. Mid-exponential phase cultures were induced with 100 μM ITPG and expression allowed to occur for 30 minutes. Cells were then pelleted. Whole cell lysate (WCL) samples were immediately boiled in SDS buffer (0.1 m Tris–HCl, pH 6.8, 4% β-mercaptoethanol, 4% SDS, 20% glycerol). For crude membrane preparations, the samples were resuspended in 1 ml PBS and subjected to a freeze/thaw cycle (−80°C/RT) three times. Unbroken cells were pelleted by centrifugation for 2 min (16,060× *g*) and the supernatant transferred to a new Eppendorf. Membranes were pelleted by centrifugation at 4°C for 1 hour (16,060× g). The pellet was then resuspended in SDS buffer and boiled for analysis by Western.

For protein detection, SDS–PAGE gels were used and then transferred to PVDF. Blots were blocked with 5% milk PBS-T and the primary antibody was Sigma's Monoclonal ANTI-FLAG M2 antibody produced in mouse (F3615). Secondary antibody was goat anti-mouse IgG-HRP (Sigma; A0168).

### Determining topology of BTP1 GtrC protein

The *gtrC*–*phoA* fusion vectors were introduced into *E. coli* NEB5a and *S. Typhimurium* 4/74. After verification by colony PCR using primers oMV1003 (CATGAAGCTTATTAATGCAGCTGGCACGAC) and oMV1006 (CGTTGGGTGATCTTTTTCGT) that strains contained the appropriate vectors, colonies were patched onto LB agar plates containing chloramphenicol and X-Phos (5-bromo-4-chloro-3-indolyl-phosphate, 40 μg ml^−1^; Melford) for detection of the blue color change.

### RNA-seq

RNA-seq was performed on *S. Typhimurium* strain D23580 cultured in 16 environmental conditions: early exponential phase, mid-exponential phase, late exponential phase, early stationary phase, late stationary phase, 25°C, NaCl shock, Bile shock, low Fe^2+^ shock, anaerobic shock, anaerobic growth, oxygen shock, nonSPI2, InSPI2, peroxide shock (InSPI2) and nitric oxide shock (InSPI2) as described (Kroger *et al*., 2012; 2013[Bibr b18],[Bibr b19]). Sequence reads were mapped to the *S. Typhimurium* D23580 genome (Genbank Accession FN424405). The BTP1 gtrA* and *gtrC* TSSs were identified in D23580 grown to ESP by dRNA-seq (Sharma *et al*., 2010). Transcript per million (TPM) values for absolute levels of gtrA* and *gtrC* gene expression were derived as described previously (Kroger *et al*., 2013).

### Preparation of phage-containing supernatants

BTP1 phage was prepared by pelleting overnight cultures of D23580, removing the supernatant and treating with a few drops of chloroform to kill any remaining bacteria. This produced phage titers with an average of 7 × 10^7^ pfu ml^−1^. To generate P22 supernatant for the experiments, a phage lysate (50 μl at 10^4^ pfu ml^−1^ of wild-type P22) was added to a 10 ml culture of exponential phase basal LT2 and growth allowed to continue overnight. Bacteria were again pelleted, the supernatant removed and treated with chloroform. This produced a supernatant containing 3.5 × 10^10^ pfu ml^−1^.

### Top agar phage assays

To create the lawn of bacteria, 100 μl of an overnight culture was mixed with 3.5 ml of molten 0.7% agar, poured onto the top of an LB agar plate and allowed to dry. Five microliters of phage-containing supernatants or dilutions thereof were spotted onto the bacterial lawns. The agar was touched with the pipette tip to indicate where drops were placed. The drops were allowed to dry before incubation overnight at 37°C.

### Verifying O-antigen cleavage by BTP1 phage

To investigate O-antigen cleavage, phage-susceptible strains were grown to overnight, pelleted and then resuspended in the phage-containing supernatants containing chloramphenicol (100 μg ml^−1^) to prevent further bacterial growth and lysis from the phage. Samples were incubated with the phage for 1 hour at 37°C before bacteria were pelleted and LPS prepared as described.

### Adsorption assay

BTP1 and P22 were diluted to a titer of 3.5 × 10^7^ pfu ml^−1^. Bacterial cells (approximately 3 × 10^8^ cfu ml^−1^) were resuspended in the diluted phage (multiplicity of infection of 1:10) and incubated at room temperature for 5 minutes. Bacteria were then pelleted and the supernatant was removed. Dilutions of the supernatant were used in the spot titer assay on a lawn of basal LT2 to determine number of phage remaining in the supernatant.
