# Supplementary material for: A BTP1 prophage gene present in invasive non-typhoidal Salmonella determines composition and length of the O-antigen of the lipopolysaccharide
Source: Mol Microbiol. 2015 Feb 11;96(2):263–75. doi: 10.1111/mmi.12933 (PMC4413052; doi:10.1111/mmi.12933)
Supplement: Supplementary file 1 — Supporting information [file mmi0096-0263-sd1.pdf]

Table S1 Oligonucleotides used in this study

oMV number	SEQUENCE (5' TO3')*	Description
oMV413	GGTAGCTCTGCAGCTTCGCATTACGAATTATAAGAAC	PstI site; Fd primer BTP1 regulatory region for cloning into CRIM
oMV439	TATTTCCGTAATATTCTCATTTGTCCTCGCCCCTGTTCTAACGTCCCATGT *GTGTAGGCTGGAGCTGCTTC*	Fam 3 gtrC end + pKD13
oMV441	TTGTCCCAAACTACTTAGCAATCAGTAGCCCCAATTGATCGGTAACAACG *ATTCCGGGGATCCGTCGACC*	Fam 3 regulatory region + pKD13
oMV442	AATTGCTGTAATTTCACCTTTGTCTACAGAAGCGTAGTACCAGAATTCAT *GTGTAGGCTGGAGCTGCTTC*	Fam 4 gtrC end + pKD13
oMV444	ATTAGTCCCTTTTCGCGCGCTATTTCCGATGAAAATGTAATCACTTTGCG *ATTCCGGGGATCCGTCGACC*	Fam 4 regulatory region + pKD13
oMV466	TTAAGACCCACTTTCACATT	tetR amplification for insertion into pCP20
oMV467	CTAAGCACTTGTCTCCTG	tetA amplification for insertion into pCP20
oMV483	CTTAATTTCGTCTTGTGTGGCACCTTGGAATTATAGGTAAAAAATGATCTACAAG *TTAAGACCCACTTTCACATT*	oafA start + tetR
oMV484	TTTTGAAATCTGCTTTTTCACTTCCTCAATAAACCACCCTGAGCCTTCTGG *CTAAGCACTTGTCTCCTG*	oafA end + tetA
oMV487	TGTGGCACCTTGGAATTATAGGTAAAAAATGATCTACAAGCCAGAAGGCTCAGGGTGGTTTATTGAGGAAGTGAAAAAGCAG	Replacement oligo for above
oMV496	TATTTCCGTAATATTCTCATTTGTCCTCGCCCCTGTTCTAACGTCCCATGT *TTAAGACCCACTTTCACATT*	Fam 3 gtrC end + tetR
oMV497	TTGTCCCAAACTACTTAGCAATCAGTAGCCCCAATTGATCGGTAACAACG *CTAAGCACTTGTCTCCTG*	Fam 3 regulatory region + tetA
oMV754	ACATGGGTGACAAGCACCCATGTAAATTTATCTTATTATCAAATG *TTAAGACCCACTTTCACATT*	BTP1 gtrC end + tetR
oMV755	ACGTAATCTGCTGCTGGCGATGGGAATTCCATGATGCACATCCG *CTAAGCACTTGTCTCCTG*	BTP1 reg region + tetA
oMV758	GCTGCTGGCGATGGGAATTCCATGATGCACATCCGCATTTGATAATAAGATAAATTTACATGGGTGCTTG	Replacement oligo for above
oMV776	TACTATAGATCTATGTTGAAGTTATTCGCTAAGTAC	BglII; P22 gtrA start
oMV777	TACTATAGATCTGTGAAATTTAATAGTAATGACAGG	BglII; P22 gtrC end
oMV780	ATTAGTGAATTCCTATTTGATTATTTTATTTCCG	EcoRI; SEN-F2 gtrC end
oMV871	ACATGGGTGACAAGCACCCATGTAAATTTATCTTATTATCAAATG *GTGTAGGCTGGAGCTGCTTC*	gtrC(402-640)^{BTP}+ pKD13 (Rv)
oMV875	CATGATGGTACCCTTTAACATGATAAAAGTCAGTGA	Acc651 site: Rv primer BTP1 regulatory region for cloning into CRIM vector
oMV894	TATGTCACTCTGTGCTGTTAAAAGATGCCATATCTAATGGTTGCA *ATTCCGGGGATCCGTCGACC*	gtrC(402-640)^{BTP} + pKD13 (Fd)
oMV897	TACTATATCGATGATAAGCTGTCAAACATGATTATCTT	ClaI; BTP1 gtrC end

	ATTATCAAATGCCCTA	
oMV898	TACTATAGATCTGTTAACTAGAGGTAGTTTAAG	BglII; extended start site BTP1 gtrC
oMV958	*CTACTTGTCGTCATCGTCCTTGTAGTC*TCTTATTATCAAATGCCCTA	Introduces C-terminal FLAG sequence onto gtrC C-terminus
oMV959	CTATATCGATGATAAGCTGTCAAACATGACTACTTGTCGTCATC	Introduces ClaI site for cloning into pLAC22 vector; to be used with oMV958 for cloning gtrC-FLAG
oMV989	CACTGTCTCCAGCTTCATCCTTTTTTTAGTTAGGGTATCTT *GTAGGCTGGAGCTGCTTCG*	wzz pKD4 vector Fd
oMV990	ATTTTACCTGTCGTAGCCGACCACCATCCGGCAAAGAAGC *CATATGAATATCCTCCTTAG*	wzz pKD4 vector Rv
oMV954	GAATTTTACAAGAGA**GCA**ATACTA**GCA**ATATTCCCAGCT	SDM** of gtrC^{BTP}
oMV955	AGCTGGGAATAT**TGC**TAGTAT**TGC**TCTCTTGTAAAATTC	SDM of gtrC^{BTP}
oMV993	CATGGTCGACGCCCCAACAAAACCACCAGG	SalI site- aa37 BTP1 gtrC
oMV994	CATGGTCGACGCCGATTTTAATATAATAGA	SalI site- aa56 BTP1 gtrC
oMV995	CATGGTCGACGCGAATATTCTTAGTATTCT	SalI site- aa76 BTP1 gtrC
oMV996	CATGGTCGACGCAATTCCATTCATGGAGTA	SalI site- aa370 BTP1 gtrC
oMV997	CATGGTCGACGCTCTTATTATCAAATGCCC	SalI site- aa640 BTP1 gtrC
oMV1003	CATGAAGCTTATTAATGCAGCTGGCACGAC	HindIII; gtrC Fd for cloning into pSK4158 (with oMV993-997)
oMV1006	CGTTGGGTGATCTTTTTCGT	phoA reverse primer to screen inserts in pSK4158
oMV1037	AGTTTTGTATTGTGCGGCCTTGCTGTACCAGGAGCACAGTAA *TTGAGCGATTGTGTAGGCTG*	TSP + pKD13 Fd
oMV1038	TAAATTTACATGGGTGCTTGTCACCCATGTTTTACAATATCA *TGATAAGCTGTCAAACATGA*	TSP + pKD13 Rv

* Restriction sites are underlined; homology with resistance cassettes are in italics
*SDM = site-directed mutagenesis. Codons creating mutations are in bold.
